# Paradoxical Anticonvulsant Effect of Cefepime in the Pentylenetetrazole Model of Seizures in Rats

**DOI:** 10.3390/ph13050080

**Published:** 2020-04-26

**Authors:** Dmitry V. Amakhin, Ilya V. Smolensky, Elena B. Soboleva, Aleksey V. Zaitsev

**Affiliations:** Sechenov Institute of Evolutionary Physiology and Biochemistry of RAS, Toreza Prospekt 44, 194223 Saint Petersburg, Russia; dmitry.amakhin@gmail.com (D.V.A.); smolensky.ilya@gmail.com (I.V.S.); soboleva.elena.1707@gmail.com (E.B.S.)

**Keywords:** epilepsy, antibiotic-induced seizures, animal models of seizures, pentylenetetrazole, Cefepime, GABAa receptor, IPSC

## Abstract

Many β-lactam antibiotics, including cephalosporins, may cause neurotoxic and proconvulsant effects. The main molecular mechanism of such effects is considered to be γ-aminobutyric acid type a (GABAa) receptor blockade, leading to the suppression of GABAergic inhibition and subsequent overexcitation. We found that cefepime (CFP), a cephalosporin, has a pronounced antiepileptic effect in the pentylenetetrazole (PTZ)-induced seizure model by decreasing the duration and severity of the seizure and animal mortality. This effect was specific to the PTZ model. In line with findings of previous studies, CFP exhibited a proconvulsant effect in other models, including the maximal electroshock model and 4-aminopyridine model of epileptiform activity, in vitro. To determine the antiepileptic mechanism of CFP in the PTZ model, we used whole-cell patch-clamp recordings. We demonstrated that CFP or PTZ decreased the amplitude of GABAa receptor-mediated postsynaptic currents. PTZ also decreased the current decay time constant and temporal summation of synaptic responses. In contrast, CFP slightly increased the decay time constant and did not affect summation. When applied together, CFP prevented alterations to the summation of responses by PTZ, strongly reducing the effects of PTZ on repetitive inhibitory synaptic transmission. The latter may explain the antiepileptic effect of CFP in the PTZ model.

## 1. Introduction

The proconvulsant action of β-lactam antibiotics was first shown in 1945 when Jonson and Walker reported seizures induced by the intraventricular administration of penicillin [1,2]. Numerous case reports later described antibiotic-induced convulsions, most often caused by cephalosporins and penicillins [3,4,5,6,7,8,9,10]. Antibiotic-induced seizures have also been reported in many animal models; a direct proconvulsant effect of drug administration [11,12,13,14] and decreased convulsive threshold to pentylenetetrazole (PTZ)-, picrotoxin-, strychnine-, or electroshock-induced convulsions [15,16,17] have been reported. The convulsant action of β-lactam antibiotics might be attributed to the inhibition of γ-aminobutyric acid (GABA) type A receptors (GABAa receptors) and the consequent suppression of inhibitory postsynaptic responses [18,19,20,21]. However, the specific mechanism of the convulsant action of many antibiotics remains unclear. Our recent study suggests that cephalosporins such as cefepime (CFP) and ceftriaxone are relatively weak blockers of GABAa receptors and that the inhibition of GABAa receptors by these antibiotics is unlikely to be the primary cause of seizures [22]. According to our data, these antibiotics could induce epileptiform activity in brain slices only in combination with the altered ionic composition of the perfusing media [22].

In this study, we focused on the effects of CFP in different experimental models of epilepsy, including the PTZ-induced seizure model and maximal electroshock threshold (MEST) test in vivo and the 4-aminopyridine model of epileptiform activity in vitro. CFP is a broad-spectrum, fourth-generation cephalosporin antibiotic, which is extensively used in clinical practice. It may have neurological side effects such as neurotoxicity [6,23,24,25], including myoclonus, nonconvulsive status epilepticus, and seizures [3,7,26,27,28,29,30]. Most clinical cases of cephalosporin-induced convulsions have been reported in patients with renal insufficiency, which led to the accumulation of the drug in the blood [3,23,31,32], although some cases have been reported in patients without kidney disease [7,25,33]. Brain disorders, including epilepsy, may also increase the risk of cephalosporin-associated convulsions [17,33,34]. Patients with brain disorders likely have a low seizure threshold due to their condition; thus, the risk of adverse effects of CFP is higher.

The proconvulsant action of cephalosporins such as CFP has been reproduced in experimental studies [14,15,17,35]. In contrast to the findings of previous studies, we found that CFP does not aggravate seizures but exhibits a pronounced antiepileptic effect by decreasing the duration and severity of convulsions and animal mortality in the PTZ-induced seizure model. This effect was specific to the PTZ model. In line with the findings of previous studies, CFP exhibited a proconvulsant effect in other models, including the maximal electroshock model and 4-aminopyridine model of epileptiform activity. The present study aimed to investigate these controversial findings and determine the potential mechanisms of effects of CFP in different epilepsy models.

## 2. Results

### 2.1. Absence of a Direct Proconvulsant Effect of CFP

First, we investigated the proconvulsant effect of CFP. None of the rats that were injected with CFP at doses of 200 and 600 mg/kg showed convulsive symptoms during 2 h of observation. Therefore, the administration of CFP was not sufficient to induce convulsions in healthy animals. Next, these animals were used in the PTZ model and MEST test.

### 2.2. An Anticonvulsant Effect of CTP on PTZ-Induced Convulsions

**Figure 1:**
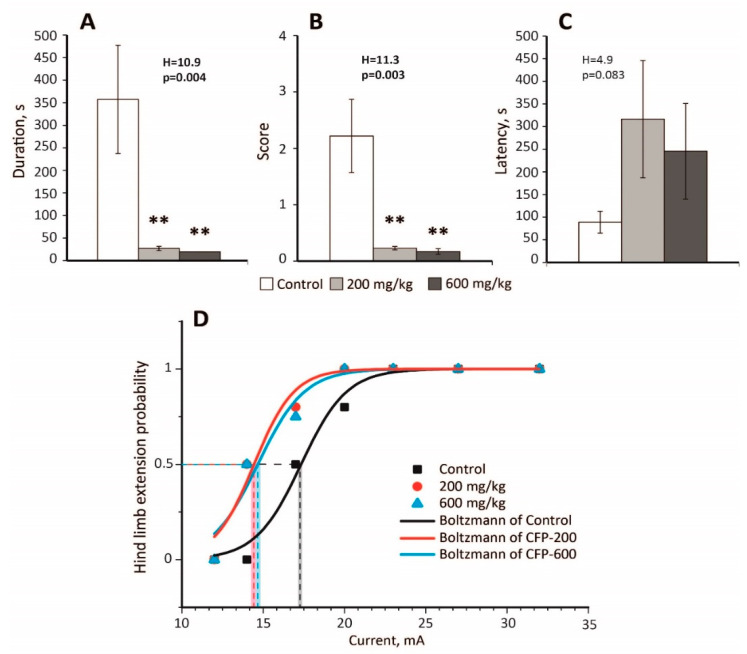
An anticonvulsant effect of cefepime (CFP) in the pentylenetetrazole (PTZ) model (**A**–**C**) and a proconvulsant effect of CFP in the maximal electroshock threshold (MEST) test (**D**). (**A**) The duration, (**B**) the mean score of severity, (**C**) the latency of PTZ-induced convulsions. H—Kruskal–Wallis one-way analysis of variance. **—significant differences from the control group (*p* < 0.01) according to the Mann–Whitney U test, which was used for post hoc comparisons. (**D**) Hind limb extension probability in the MEST test. Experimental results are approximated with Equation (1), and the corresponding EC_50_ values are marked with dashed lines; shadow represents the standard error of parameter estimates.

In the PTZ model (70 mg/kg), pretreatment with CFP at doses of 200 and 600 mg/kg significantly decreased the duration of convulsions (Figure 1A) and their severity (Figure 1B) in rats (*n* = 9 animals in each group). It also prevented the development of status epilepticus in both experimental groups that received CFP (CFP-200 and CFP-600 groups); 0% rats had status epilepticus in the CFP-200 and CFP-600 groups, whereas 33% rats had status epilepticus in the control group (*p* = 0.03, Fisher’s exact test). No rats died in the CFP-200 and CFP-600 groups, whereas the mortality rate was 56% in the control group (*p* = 0.002, Fisher’s exact test). The latency of convulsions was similar in all the groups (Figure 1C).

Despite being a competitive antagonist of GABAa receptors [35], CFP exhibited a strong antiepileptic effect in the PTZ model.

### 2.3. Decreased Seizure Threshold in the MEST Test Following CFP Administration

Next, we investigated whether CFP affected the seizure threshold in the MEST test. Table A1 presents the results of the experiments. In both CFP-200 and CFP-600 groups, a significant decrease in the seizure threshold was observed (Figure 1D). By fitting the curves with the Boltzmann equation, we found that EC_50_ value in the control group (17.3 ± 0.3 mA) was larger than those in the CFP-200 group (14.4 ± 0.4 mA, *p* < 0.001, *t*-test with Bonferroni correction) and CFP-600 group (14.6 ± 0.4 mA, *p* < 0.001, *t*-test with Bonferroni correction). Thus, CFP treatment had a proconvulsant effect and reduced the seizure threshold in the MEST test by 16–17%.

### 2.4. The Effect of CFP in the 4-Aminopyridine Model of Epileptiform Activity

**Figure 2:**
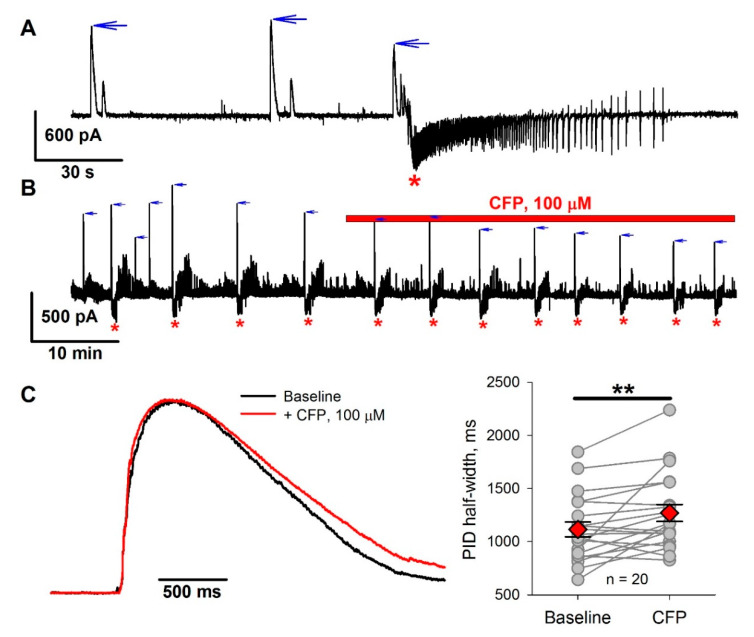
The effect of cefepime (CFP) in the 4-aminopyridine model. (**A**) A representative voltage clamp recording of epileptiform activity in entorhinal cortex slices. Vhold = −27 mV, which is about halfway between the reversal potentials of glutamate- and γ-aminobutyric acid (GABA)-mediated currents. Two main types of epileptiform events can be observed: GABA-mediated preictal discharges (PIDs), which manifest themselves as positive currents at −27 mV (blue arrows), and seizure-like events (SLEs) (red asterisks), during which both positive (GABA-mediated) and negative (glutamate-mediated) currents are observed. (**B**) A representative voltage clamp recording, illustrating the effect of bath application of CFP. Note an increase in SLE frequency and a decrease in PID amplitude after CFP administration. **(C)** The effect of CFP on PID duration. Left panel: normalized and averaged current, recorded during PIDs. Right panel: the graph shows changes in the PID half-width. ** *p* < 0.01 (paired *t*-test).

**Figure A1:**
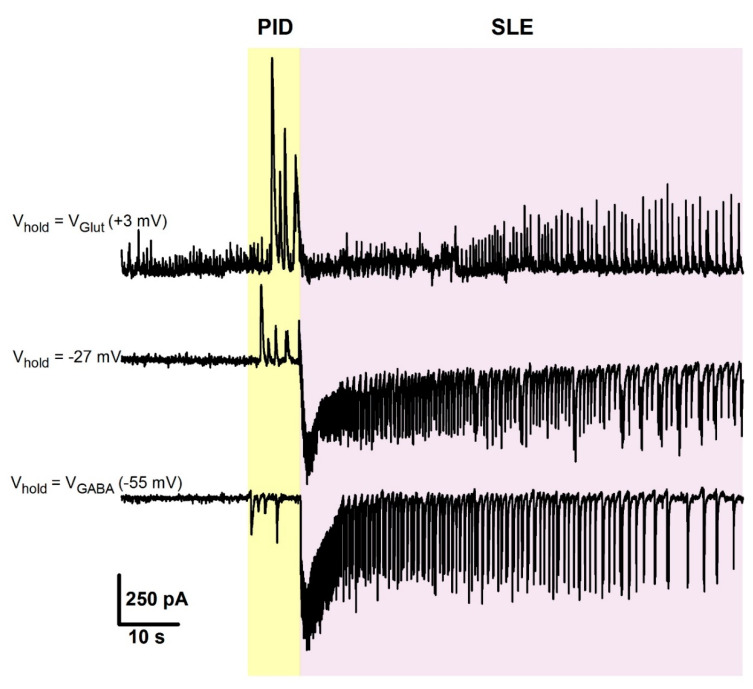
Representative voltage-clamp recordings of synaptic activity during preictal discharges (PIDs) and seizure-like events (SLEs), obtained from the same cell at different holding potentials. Note, that currents corresponding to PIDs and IIDs are reversed at different voltages. At +3 mV, a reversal potential for glutamate-mediated current, only the GABA-mediated component is present and is most prominent during PIDs. At −55 mV, a reversal potential for GABA-mediated current in these recording conditions, only glutamate-mediated component is present, and during the PID phase, its contribution is low.

We also tested the effect of CFP on epileptiform activity in the 4-aminopyridine model using entorhinal cortex slices. Cerebrospinal fluid concentrations of cephalosporins in healthy animals are an order of magnitude lower than blood concentrations [31,36]. Therefore, we used CFP at a concentration of 100 µM, which is comparable with that used in our in vivo experiments. Perfusion of slices with proepileptic solution resulted in epileptiform activity, which was described in detail in our previous studies [37,38,39,40], and consisted of to two major forms: GABA-mediated preictal discharges (PIDs) and subsequent seizure-like events (SLEs) which are mediated by both GABA and glutamate (Figure 2A and Figure A1). SLEs in slices are considered to be an analog of seizures in the brain [41,42]. We investigated the effect of CFP on the frequency of SLEs (Figure 2B). The administration of CFP resulted in a 32% increase in SLE frequency, from 2.2 ± 0.2 to 2.9 ± 0.2 mHz (*n* = 15 slices, *p* < 0.01, paired *t*-test). As CFP is thought to affect GABA-mediated synaptic transmission, we also analyzed the effect of CFP on PIDs. CFP did not affect the frequency of PIDs (7.1 ± 0.6 mHz before administration vs. 8.6 ± 1.0 mHz after administration, *n* = 19, *p* = 0.08, paired *t*-test) but caused a 19% reduction in the peak amplitude of PIDs, from 716 ± 85 to 577 ± 90 pA (*p* < 0.01). It also increased the PID half-width by 14% (Figure 2С).

Therefore, CFP had a proepileptic effect in the 4-aminopyridine model.

### 2.5. Occlusion of the Effects of PTZ and CFP on Inhibitory Synaptic Transmission

Next, we investigated the potential antiepileptic mechanism of action of CFP in the PTZ model. Unfortunately, we were not able to reproduce PTZ-induced epileptiform activity in slices of the entorhinal cortex. The application of PTZ in a dose of 400 µM, which is comparable with that used in our in vivo experiments, did not produce epileptiform activity in slices which is most likely due to the transection of neuronal connections. Therefore, we could not directly study the effect of CFP on the epileptiform activity induced by PTZ.

As both PTZ and CFP target GABAa receptors, a possible explanation for the antiepileptic effect of CPF in the PTZ model would be the occlusion of the effects of these drugs, so we investigated the effect of these drugs on inhibitory currents. To test this possibility, we implemented a one-way analysis of variance (ANOVA) and compared the relative effects of CFP (100 µM), PTZ (400 µM), and their combination on the properties of pharmacologically isolated evoked inhibitory postsynaptic currents (eIPSCs).

**Figure 3:**
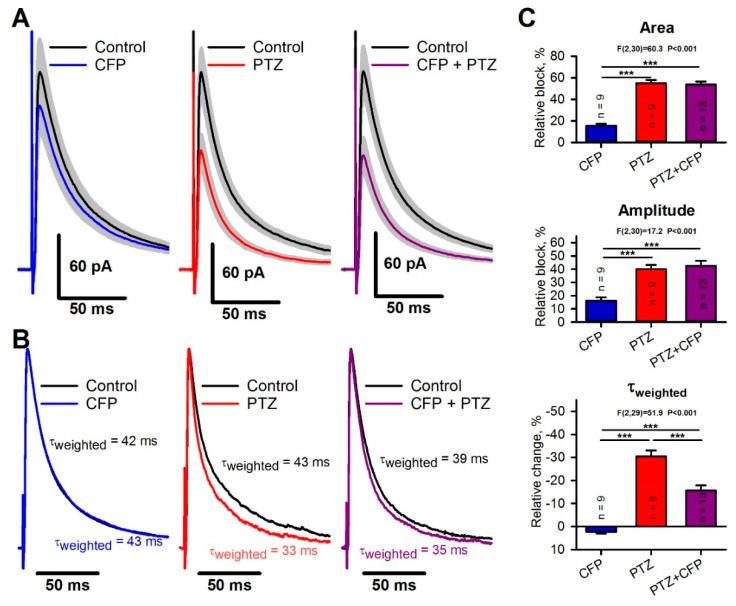
The effects of cefepime (CFP), pentylenetetrazole (PTZ), and their combination on γ-aminobutyric acid type a (GABAa) receptor-mediated evoked inhibitory postsynaptic currents (eIPSCs). (**A**) Averaged recordings of eIPSCs. The gray area represents the standard error of the mean. (**B**) Representative examples of normalized eIPSCs. Note that PTZ alone induces a more prominent decrease in the current decay constant than PTZ in combination with CFP. (**C**) The bar diagrams showing the effects of drugs on the eIPSC area, peak amplitude, and weighted time constant. F-statistics and corresponding *p*-values are shown for a one-way analysis of variance (ANOVA). Asterisks indicate the significance according to the Tukey test, used for post hoc comparisons (*** *p* < 0.001).

CFP induced weak blockade of the GABA-mediated current. The average eIPSC amplitude and area both decreased by the same value of 16 ± 2% (paired *t*-test, *p* < 0.05; Figure 3A,C), but the weighted time constant of the current decay increased from 41 ± 6 ms to 44 ± 7 ms. The average increase was 4 ± 2% (*P* = 0.03, Figure 3B,C). PTZ reduced the eIPSC amplitude by 40 ± 3% (*p* < 0.001) and the area by 55 ± 3% (*p* < 0.001, Figure 3A,C). In contrast to CFP, PTZ strongly accelerated the decay kinetics and decreased the weighted time constant from 41 ± 5 ms to 28 ± 3 ms (Figure 3B, middle panel). The average decrease was 30 ± 3% (*p* < 0.001, Figure 3C). By fitting the decay phase of the response with the biexponential function [Equation (3)], we revealed that PTZ blocks the slow component of the inhibitory current to a greater extent than the fast one (63 ± 3% vs. 23 ± 4%, *p* < 0.01, paired *t*-test).

The Tukey post hoc tests revealed that the combination of CFP and PTZ resulted in the same blockade of eIPSC amplitude and area as the administration of PTZ (Figure 3A,C). However, the combination of CFP and PTZ affected the kinetics of response less than PTZ alone. The changes in the weighted time constant were smaller (16 ± 2%, Figure 3B,C) than those after the administration of PTZ alone. Therefore, CFP decreases the ability of PTZ to block the slow components of the GABA-mediated postsynaptic current.

**Figure 4:**
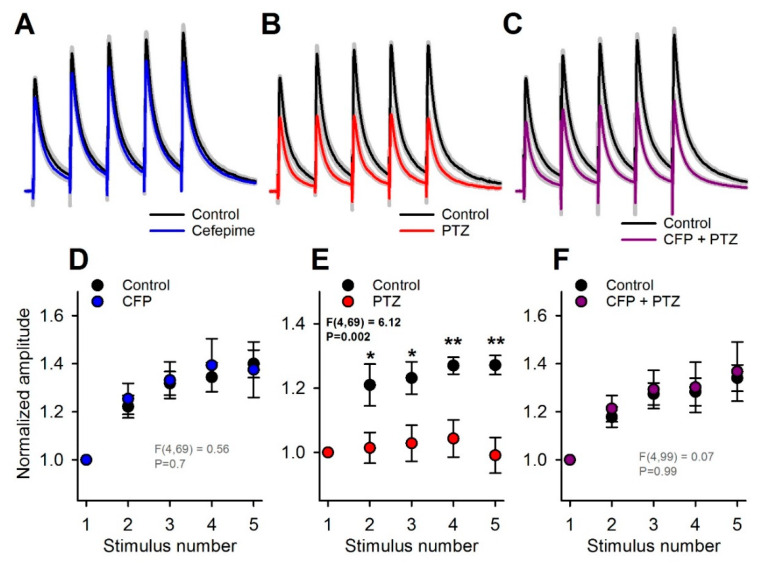
The effects of cefepime (CFP) and pentylenetetrazole (PTZ) on the train of γ-aminobutyric acid type a (GABAa) receptor-mediated evoked inhibitory postsynaptic currents (eIPSCs). (**A**–**C**). Averaged recordings of trains of eIPSCs. The gray area represents the standard error of the mean. The recordings represent the effect of CFP (**A**), PTZ (**B**), and their mixture (**C**) on the control response. All averaged responses were normalized to the amplitude of the first peak of control response. (**E**,**F**) Averaged peak amplitudes of eIPSCs, normalized to the peak amplitude of the first response in the train. The effect of drugs on the summation of eIPSCs was tested using two-way repeated measures ANOVA. The results indicate that PTZ strongly alters summation (**E**), while CFP (**D**) or a mixture of CFP and PTZ (**F**) do not. Asterisks indicate the significant difference between eIPSC amplitudes according to the Tukey’s test, used for post hoc comparisons (* *p* < 0.05; ** *p* <0.01).

As this effect might accumulate during the repetitive synaptic activation of GABAa receptors, we analyzed the effect of PTZ and CFP on responses to multiple stimuli. For this purpose, we applied trains of five stimuli at 10 Hz. In the control, the response amplitude gradually increased within the train, mostly due to temporal summation. As CFP exerted a feeble effect on the kinetics of eIPSC, the summation of these responses was not disturbed by CFP administration (*n* = 7, repeated-measures two-way ANOVA, F_4,69_ = 0.56, *p* = 0.70; Figure 4A,D). In contrast, PTZ significantly altered the temporal summation of the responses (*n* = 7, F_4,69_ = 6.12, *p* = 0.002, Figure 4B,E), decreasing the normalized peak amplitude of the fifth eIPSC from 1.27 ± 0.08 to 0.99 ± 0.06 (*n* = 7, Tukey post hoc test, Figure 4E). Thus, the blockade of the slow component of the response by PTZ resulted in a disturbed summation during repetitive stimulation. The combination of CFP and PTZ did not affect the summation of the responses (*n* = 10, F_4,99_ = 0.07 *p* = 0.99; Figure 4C,F). Thus, the administration of CFP helped to preserve the summation of the inhibitory responses.

These results indicate that the protective effect of CFP against PTZ-mediated blockade of GABAa receptors is most prominent during repetitive stimulation. It is generally assumed that due to higher connectivity, the frequency of synaptic events is higher under in vivo conditions than in in vitro slice preparations [43]. Thus, it is highly likely that this protective effect of CFP would be manifested under in vivo conditions and promote the antiepileptic effect of CFP in the PTZ model.

## 3. Discussion

In the present study, we assessed the proconvulsant effect of different doses of CFP in rats. We also used two in vivo tests to estimate the convulsive liability of CFP-treated rats and studied the effect of CFP on epileptiform activity in the 4-aminopyridine in vitro model. We found that the administration of CFP at relatively high doses of 200 and 600 mg/kg was not sufficient to induce convulsions. As reported previously for another first-generation cephalosporin, cefazolin [15], only extremely high doses (3 g/kg, intravenously) resulted in wild running, jumping, rolling, and finally, death of mice. Lower doses (0.5–2 g/kg) did not produce convulsive symptoms on their own [15]. However, the absence of motor convulsions cannot exclude the possibility that CFP induces epileptiform activity in the brain. For example, Tanaka et al. revealed abnormal electroencephalogram (EEG) spikes in mice administered CFP at a dose of 500 mg/kg, with no behavioral signs [17].

Only a few studies have estimated the convulsant effect of CFP in animal epilepsy models [17,22,35,44]. Despite the fundamental differences between the three implemented models of epileptic seizures, in MEST and 4-AP model, CFP exhibited a proconvulsant effect, which is in line with multiple clinical reports of its neurotoxicity in patients [28]. During the MEST test, rats pretreated with CFP showed a modest decrease in the convulsive threshold. Similar results were observed in a study of electroshock-induced convulsions in mice [17]. CFP at a dose of 500 mg/kg, but not 250 mg/kg, increased the severity of seizures induced by electroconvulsive shock at low-stimulus currents [17].

The PTZ model yielded the opposite outcome. The PTZ-induced convulsion test is considered a useful screening method for validating the proconvulsive liability of antibiotics [45]. PTZ is an antagonist of GABAa receptors at IC_50_ values of 0.6–2.2 mM [46,47]. Thus, PTZ decreases the effectiveness of GABA-mediated inhibition promoting increased network excitability. In the present study, CFP pretreatment resulted in a significant reduction in the duration and severity of seizures in the PTZ test. According to previous studies, different cephalosporins have diverse effects on PTZ-induced convulsions [15,17,48,49,50]. Cefazolin was shown to exhibit a significant proconvulsant effect at a dose of 800 mg/kg, but not 400 mg/kg, in rats and mice [49,50]. Another study in mice revealed that when cefazolin at a dose of 500 or 1000 mg/kg was injected 5 min before the administration of subconvulsive doses of PTZ (40 or 60 mg/kg), it enhanced the convulsant ability of PTZ. There was also a significant increase in the mortality rate [15]. In striking contrast, ceftriaxone (200 and 400 mg/kg) was found to have protective effects on PTZ-induced convulsions [48], which was explained by the activation of the glutamate transporter GLT1 [48,51]. The authors demonstrated an anticonvulsant effect with both EEG recordings and behavioral observations [48]. In another reported PTZ test, CFP administration (500 mg/kg) did not change the mean seizure stage and a lower dose of CFP (250 mg/kg) administration prevented convulsions. The authors concluded that CFP at doses of 250 and 500 mg/kg had no effect on PTZ-induced convulsions in mice [17]. Since the authors used a low dose of PTZ (40 mg/kg) and the mean seizure stage was very low (0.2), the possible anticonvulsant action of CFP in the study [17] cannot be excluded.

Although it is generally assumed that convulsions induced by β-lactam antibiotics are associated with the inhibition of GABA-mediated responses [18,19,20,21], the ability of CFP to antagonize GABAa receptors might not be the primary mechanism of its proconvulsant effect in clinical practice. Estimations of the strength of GABAa receptor blockade by cephalosporins [35] indicated that cefazolin had more than an order of magnitude lower IC_50_ than CFP used in the present study. In our previous study [22], extremely high concentrations of CFP (>1 mM) in artificial cerebrospinal fluid were required to induce substantial blockade of the GABA-mediated postsynaptic current. Even at high concentrations, CFP failed to exhibit consistent epileptiform activity in vitro [22].

Previous studies have reported that both PTZ and CFP are relatively weak antagonists of GABAa receptors, with a predominantly competitive mechanism of action [35,46]. For α1β2γ2 GABAa receptors, CFP inhibited GABA (ED_20_)-induced currents, with an IC_50_ of about 2.6 mM [35]. In contrast, about 0.6 mM PTZ was required to achieve half-maximal inhibition of GABA ED_30_-induced currents [46]. These data indicated that PTZ is at least a five times more potent antagonist of GABAa receptors than CFP.

Using the whole-cell patch-clamp method, we demonstrated that the potential mechanism responsible for the observed antiepileptic effect of CFP might be a molecular interaction of CFP and PTZ at GABAa receptors, as has been reported for several other pharmacological agents [47,52]. In our previous study [22] and in the present study, we demonstrated the ability of CFP to potentiate late components of GABA-mediated postsynaptic currents. As CFP potentiates the slow component of the postsynaptic current and PTZ blocks it to a greater extent than the fast ones, the most likely explanation of the observed occlusion between CFP and PTZ is that these two effects cancel each other out, resulting in a less pronounced effect of PTZ in the presence of CFP. Our results indicated that even when CFP was administered at high doses, its concentration in the extracellular medium in the brain was sufficient to decrease the seizure threshold and affect the ability of PTZ to induce seizures. Still, it was not enough to induce substantial blockade of GABAa receptors on its own. The exact molecular mechanism of such cross-interactions between these two ligands of GABAa receptors remains unknown.

In the present study, we also revealed that CFP had a proepileptic effect in the 4-aminopyridine model. This fact is in agreement with the observed changes in GABA-mediated currents during PIDs in the acute 4-aminopyridine model. It is hypothesized that at some point in time before SLE initiation most pyramidal cells in the slice may experience an activity-dependent collapse of the chloride gradient, which will render GABAergic neurotransmission excitatory [53]. The chloride loading is enhanced in the case of impaired chloride extrusion capacity [54,55]. Under such conditions, the prolonged kinetics of GABAergic currents may contribute to seizure initiation by accelerating the onset of such activity-dependent collapse and prolonging the GABA-mediated event which launches the epileptic discharge. In in vivo models, like PTZ, the situation might be different: the initiation of seizures is more likely to be attributed to PTZ-induced blockade of GABAergic neurotransmission, rather than to its activity-dependent failure. As the increased activity of GABAergic interneurons is hypothesized to be the primary cause of seizure onset in in vitro models and in some forms of seizures in vivo [53,56,57,58,59], the ability of CFP to prolong the kinetics of GABA-mediated currents can potentially outweigh its ability to inhibit them, which might lead to more efficient recruitment of glutamatergic neurons and result in more frequent SLE initiation. This effect can provide an alternative mechanism for the proepileptic action of CFP.

## 4. Materials and Methods

### 4.1. Animals

Male Wistar rats aged 6 wk (*n* = 61) were used in this study for in vivo experiments, and 3-wk-old rats (*n* = 18) were used for in vitro experiments. The animals were kept under standard conditions with free access to food and water. All animal procedures followed the guidelines of the European Community Council Directive 86/609/EEC and approved by the Sechenov Institute of Evolutionary Physiology and Biochemistry Bioethics Committee (Ethical permit number 13-k-a, 15 February 2018).

### 4.2. CFP Administration

CFP (cefepime hydrochloride monohydrate, Sigma-Aldrich Corporation, St. Louis, MO, USA) was administered i.p. There were three groups, as follows: control (received a saline injection), CFP-200 (received CFP in the dose of 200 mg/kg), and CFP-600 (received CFP in the dose of 600 mg/kg). In the groups, CFP or saline was administered at an injection volume of 2 mL/kg of body weight. All the rats in each group were monitored for 2 h after the injection to investigate the direct proconvulsive action of CFP.

### 4.3. Maximal Electroshock Threshold (MEST) Test

Two hours after the administration of CFP, the MEST test was used to estimate its effect on the convulsions threshold in the three groups (control (*n* = 11), CFP-200 (*n* = 11), and CFP-600 (*n* = 12)). A Pulse Generator ECT Unit 57800 (Ugo Basile, Gemonio, VA, Italy) was used to administer the rectangular current steps via aural electrodes (ear clips). The current intensity varied between 12 and 32 mA; the pulse duration was 0.7 s, and the step frequency and step width were 200 Hz and 0.7 ms, respectively. The MEST was considered the minimal current that resulted in full hind limb extension using the up-and-down staircase method [60,61,62]. Thus, the first rat received current with moderate intensity (27 mA), the second rat then received a lower (23 mA) or higher (32 mA) current, depending on the presence or absence of full hind limb extension in the first rat. To ensure that the intervals in the log scale were equal to 0.07, the current steps were as follows: 12, 14, 17, 20, 23, 27, and 32 mA. The probability of convulsions was calculated as the ratio of the number of rats with full hind limb extension after the administration of the current to the total number of rats that received the current. To calculate this probability, we assumed that if full hind limb extension occurred in an animal after a specific current (tonic hind limb extension in Table A1), it would occur in the same animal after higher currents (assumed extension in Table A1). Equally, if full hind limb extension did not occur after a particular current (lack of extension in Table A1), it would not occur in the same animal after lower currents (assumed lack of extension in Table A1). The Boltzmann equation was used to fit the dependence of full hind limb extension probability from the value of presented current:

(1) $$\mathrm{Probability}\;\left(I;Slope,EC50\right)=\frac{1}{1+\exp\left(\frac{\mathrm{EC}50-I}{\mathrm{Slope}}\right)},$$

where *EC50* was the current with a 50% probability of convulsions and *Slope* was the slope of the sigmoid.

### 4.4. PTZ Test

Two hours after CFP administration (this time interval was selected based on the pharmacokinetic properties of CFP and other cephalosporins [8,63]), the rats in each group (*n* = 9 in each group) were i.p. injected with PTZ (Sigma-Aldrich, 70 mg/kg), a GABAa receptor antagonist dissolved in saline. The animals were then placed in individual transparent cages for video recording for 30 min. Convulsions were rated according to the modified Racine scale [64] for PTZ-induced convulsions [65,66,67]. Thus, a score of 1 denoted single to repeated myoclonic jerks; a score of 2 denoted partial clonic seizure in a sitting position; a score of 3 denoted generalized convulsions, including clonic and/or tonic-clonic seizures while lying on their belly; and a score of 4 denoted generalized convulsions, including pure tonic seizures with hind limb extension and/or tonic-clonic seizures while lying on their sides that could start with wild running. The convulsive behavior of each rat was described according to the following parameters: the total duration of the convulsions, the latency to the convulsions (time to the first myoclonic jerks), and the average score of the convulsions during the observation period. The latter was calculated as the average value of the maximal score during each 30-s interval within 30 min of observation. The mean values were calculated for all the groups, as well as the mortality rate and the number of rats that had status epilepticus, which was defined as convulsions that lasted for more than 10 min.

### 4.5. Brain Slice Preparation

The rats were sacrificed via decapitation, and their brains were removed rapidly. The brain slice preparation method has been described previously [37]. A vibrating microtome (Microm HM 650 V; Microm, Germany) was used to cut horizontal 300-μm-thick slices that contained the entorhinal cortex and hippocampus. Artificial cerebrospinal fluid with the following composition (in mM) was used: 126 NaCl, 24 NaHCO_3_, 2.5 KCl, 2 CaCl_2_, 1.25 NaH_2_PO_4_, 1 MgSO_4_, and 10 dextrose. The artificial cerebrospinal fluid was aerated with a gas mixture of 95% O_2_ and 5% CO_2_. All chemicals used for the preparation of the solutions were purchased from Sigma-Aldrich, unless stated otherwise. Three to four slices per rat were used for patch-clamp recordings.

### 4.6. Whole-Cell Patch-Clamp Recordings

The recordings were performed at 30 °C. Pyramidal neurons in the deep layers of the medial entorhinal cortex were visualized using a Zeiss Axioscop 2 microscope (Zeiss, Oberkochen, Germany) equipped with differential interference contrast optics and a video camera (Grasshopper 3 GS3-U3-23S6M-C; FLIR Integrated Imaging Solutions Inc., Wilsonville, OR, USA). Patch electrodes (3–5 MΩ) were pulled from borosilicate glass capillaries (Sutter Instrument, Novato, CA, USA). A cesium methanesulfonate-based pipette solution (composition in mM: 127 CsMeSO_3_, 10 NaCl, 5 EGTA, 10 HEPES, 6 QX314, 4 ATP-Mg, and 0.3 GTP; pH adjusted to 7.25 with CsOH) was used. Whole-cell recordings were performed using a Model 2400 (AM-Systems, Sequim, WA, USA) patch-clamp amplifier and an NI USB-6343 A/D converter (National Instruments, Austin, TX, USA) using WinWCP 5 software (University of Strathclyde, Glasgow, U.K.). The data were filtered at 10 kHz and sampled at 20 kHz. In all cells included in the sample, access resistance was less than 15 MΩ and remained stable (≤20% increase) across the experiment. The liquid junction potential was compensated for offline for the voltage-clamp recordings by subtracting 7 mV.

The synaptic responses were evoked extracellularly. A stimulating twisted nichrome electrode was placed in the same layer as the recorded neuron at a distance of 100–200 μm. Postsynaptic GABAa receptor-mediated currents were recorded at 0 mV in the presence of 10 μM DNQX (Tocris Bioscience, Bristol, UK) and 50 µM AP-5 (Tocris Bioscience). The amount of charge transferred during the eIPSC (i.e., the response area) was calculated as the area under the current-time plot (0–750 ms from the stimulus).

CFP (100 μM) or PTZ (400 μM) was bath applied either separately or in combination. The relative block of the GABAa receptor-mediated current was calculated using Equation (2):

(2) $$\mathrm{Block}=100\%\times \frac{A_{baseline}-A_{block}}{A_{baseline}},$$

where $A_{baseline}$ and $A_{block}$ were the average amplitudes or areas of 10–15 evoked IPSCs, which were recorded before and 10 min after drug administration.

The kinetics of GABAa receptor-mediated eIPSCs were estimated using a nonlinear regression analysis of the decay phase (10–90%). The biexponential function below was utilized:

(3) $$I\left(t;A_{fast},\tau_{fast},A_{slow},\tau_{slow}\right)=A_{fast}*\exp\left(-\frac{t}{\tau_{fast}}\right)+A_{slow}*\exp\left(-\frac{t}{\tau_{slow}}\right),$$

where $A_{fast}$ and $\tau_{fast}$ were the amplitude and time constant of the fast-decaying component, respectively, and *A_slow* and *τ_slow* were the amplitude and time constant of the slow-decaying component, respectively. To estimate the effect of the drugs on the fast and slow components of the response, we first fitted each decay in the pair using Equation (3) with unconstrained coefficients. Next, for each pair of responses, the average $\tau_{fast}$ and $\tau_{slow}$ were calculated, and both decays were refitted with Equation (2) with fast and slow time constants being set equal to averaged ones. The relative block of the fast or slow component of the response was calculated using Equation (2), where $A_{baseline}$ and $A_{block}$ were the average amplitudes of the corresponding components before and after drug administration, respectively.

The weighted time constants were calculated using the following formula:

(4) $$\tau_{weighted}=\frac{\tau_{fast}*\;A_{fast}+\tau_{slow}*A_{slow}}{A_{fast}+A_{slow}}$$

### 4.7. 4-Aminopyridine In Vitro Model of Epileptiform Activity

Epileptiform activity in rat brain slices was induced using a pro-epileptic solution [37], containing the following (in mM): 126 NaCl, 24 NaHCO_3_, 2.5 KCl, 2 CaCl_2_, 1.25 NaH_2_PO_4_, 0.25 MgSO_4_, 10 dextrose, 0.05 4-aminopyridine, which, by blocking voltage-gated potassium channels increases action potential duration, resting membrane potential and input resistance, thus leading to increased network excitability [68]. The stable generation of epileptiform activity in the entorhinal cortex was achieved after 15–30 min of perfusion. When a stable generation of both PIDs and SLEs was achieved, we recorded the baseline activity until 3–5 SLEs were accumulated. After that CFP (100 µM) was administered, and the recording proceeded for the next 30–45 min. Only those epileptiform events which were registered after 7 min of perfusion with CFP were included in analysis.

### 4.8. Statistics

The data analysis was performed using custom software written in Wolfram Mathematica 10 (Wolfram Research, Champaign, IL, USA). Sigmaplot 14 (Systat Software Inc., San Jose, CA, USA) was used for the statistical analysis and graphical representation of the results. Dixon’s *Q*-test (at the 95% confidence level) was used to reject outliners. The Kolmogorov–Smirnov test was employed for the evaluation of the normality of sample data. The equality of variance was assessed using the Levene median test. For data that had a normal distribution and passed an equal variance test, the statistical significance was assessed using a paired Student’s *t*-test, one-way ANOVA, or two-way repeated measures ANOVA. For data that did not pass the normality test, the Kruskal–Wallis one-way ANOVA on ranks and Mann–Whitney rank sum test were used, where appropriate.

## Abbreviations

CFP	Cefepime
GABA	γ-Aminobutyric Acid
IPSC	Inhibitory Postsynaptic Current
MEST	Maximal Electroshock Threshold
PTZ	Pentylenetetrazole
PID	Preictal Discharge
SLE	Seizure-Like Event

## Appendix A

**Table A1:** Results of the MEST test in three groups of rats.

mA	1	2	3	4	5	6	7	8	9	10	11	12	Probability
**Control**													
12			-				-		-	-			0%
14			-				0		0	-			0%
17			-			x		x		0			50%
20			0		x	*		*			x		80%
23		x		x	*	*		*			*		100%
27	x	*		*	*	*		*			*		100%
32	*	*		*	*	*		*			*		100%
**CFP-200**													
12				-				0	-				0%
14				-			x		0		x		50%
17				0		x	*			x	*		80%
20			x		x	*	*			*	*		100%
23		x	*		*	*	*			*	*		100%
27	x		*		*	*	*			*	*		100%
32	*		*		*	*	*			*	*		100%
**CFP-600**													
12					-			-		-	0		0%
14					-			0		0		x	25%
17					0		x		x			*	75%
20				x		x	*		*			*	100%
23			x	*		*	*		*			*	100%
27		x	*	*		*	*		*			*	100%
32	x	*	*	*		*	*		*			*	100%

Each vertical column represents the results for one rat from the corresponding group. “x”—tonic hind limb extension, “0”—lack of extension, “*”—assumed extension, “-”—assumed lack of extension.
